# Naltrexone, Naloxone, Morphine, and Fentanyl Pharmacologically Chaperone a Mutant μ‐Opioid Receptor via an Endoplasmic Reticulum Exit Site‐Dependent Pathway

**DOI:** 10.1002/prp2.70315

**Published:** 2026-09-17

**Authors:** Stephen N. Grant, Matthew J. Mulcahy, Henry A. Lester

**Affiliations:** ^1^ Division of Chemistry and Chemical Engineering California Institute of Technology Pasadena California USA; ^2^ Division of Biology and Biological Engineering California Institute of Technology Pasadena California USA

**Keywords:** endoplasmic reticulum, microscopy, opioids, μ‐Opioid receptor

## Abstract

Opioid receptor antagonists increase the plasma membrane density of μ‐opioid receptors (MOR) in vivo, thereby inducing “supersensitivity” to opioid agonists. This phenomenon increases the risk of overdose in patients that were being treated with naltrexone (Ntx), a popular MOR antagonist. Although there are many studies on the effects of ligands on the plasma membrane density of MORs, their effects on MOR trafficking from the endoplasmic reticulum (ER) via endoplasmic reticulum exit sites (ERES) have not been investigated. Using fluorescently tagged Sec24 to visualize ERES and a highly ER‐retained μ‐opioid receptor (MOR) point mutant, MOR[N190K], we measured ERES levels after incubation in various opioid receptor ligands. Data from sensitized emission Förster resonance energy transfer (FRET) show that MOR[N190K] interacts with Sec24D. We observe that Ntx, naloxone, morphine, and fentanyl increase the fraction of the cytoplasm occupied by ERES. In contrast, buprenorphine, methadone, and two allosteric modulators have no substantial effect on ERES levels. Mutating S375, an important phosphorylation site for MOR internalization, decreased morphine and fentanyl's pharmacological chaperoning abilities but did not affect Ntx's pharmacological chaperoning abilities. Ntx did not change intracellular cyclic adenosine monophosphate (cAMP) concentrations, so global trafficking is not expected to change. We also find that antagonist‐induced pharmacological chaperoning depends on retrotransport from the Golgi as brefeldin A (BFA) treatment prevented pharmacological chaperoning. These data reveal new understandings of how opioid ligands change MOR trafficking from the ER and can help us better understand the pathophysiology of opioid use disorder.

**Abbreviations**
BFA: brefeldin A
cAMP: cyclic adenosine monophosphate
CNS: central nervous system
COPI: Coat Protein I
ER: endoplasmic reticulum
ERES: endoplasmic reticulum exit sites
FRET: Förster resonance energy transfer
Me‐Ntx: N‐methylnaltrexone
MOR: μ‐opioid receptor
Ntx: naltrexone

## 1 Introduction

The MOR is the primary target for most clinically used analgesic opioids [1, 2]. Understanding how the MOR enters and exits the plasma membrane is critical to understanding the causes of opioid use disorder [1, 3, 4, 5, 6, 7]. Chronic application of certain agonists results in decreased MORs on the plasma membrane [8]. In the prevailing view (not challenged in this study), these agonists cause phosphorylation at the C‐terminus via G‐protein receptor kinases, which induce β‐arrestin binding and endocytosis [9]. The opioid peptides and some synthetic alkaloid agonists, like fentanyl, broadly cause rapid endocytosis [10]. In turn, researchers have tried to develop biased agonists that maintain analgesic properties while reducing their ability to internalize receptors, hopefully also leading to a reduction in their dependence and tolerance potential [11, 12].

In contrast, chronic doses of the two most common opioid antagonists, naltrexone (Ntx) and naloxone [13], increase the number of MORs on the cell surface. This upregulation of surface levels of opioid receptors in response to antagonists causes supersensitivity in vivo [14, 15]. Supersensitivity is a health concern because antagonists are prescribed for individuals recovering from opioid use disorder. However, if antagonists are not present and the patient relapses to opioid use, then supersensitivity will increase the risk of overdose [16, 17]. These increased MOR levels may last weeks after the final administration of the antagonist [18]. Preventing opioid receptor surface level upregulation will decrease the health risks involved in prescribing opioid antagonists.

Several mechanisms could underlie upregulation. Since certain agonists induce endocytosis, perhaps the prevention of basal MOR activation decreases basal endocytosis and increases MOR plasma membrane levels [4, 10, 19, 20]. Alternatively, after endocytosis, the antagonists could be increasing the recycling of the receptors. There is evidence that some opioid ligands can bias toward the recycling pathway [21]. There is also a cAMP hypothesis: recent work demonstrated that increases in intracellular [cAMP] coincide with global increases in protein trafficking [22]. MOR activation decreases [cAMP], so decreasing MOR activity may increase [cAMP] and trafficking [23].

This study examines the pharmacological chaperoning hypothesis [24]. Pharmacological chaperoning occurs when a pharmacophore binds to a nascent protein in the ER and promotes proper folding, thus aiding its exit from the ER [25]. Pharmacological chaperoning participates in therapeutic approaches when sub‐optimal protein levels reach the plasma membrane [25, 26, 27, 28]. Agonists and antagonists can chaperone in various systems [29, 30].

Research investigating pharmacological chaperoning has not yet examined key early events in MOR surface expression. Others have investigated other GPCRs, such as the V2‐vasopressin receptor, the GnRH receptor, and the δ‐opioid receptor [24, 31, 32]. The difficulty in studying the MORs is partly because MORs traffic rather efficiently to the plasma membrane [1]. Therefore, we have investigated the MOR[N190K] mutant, which is substantially retained in the ER [33]. We established that Ntx and naloxone could increase the plasma membrane density of this mutant. In previous experiments with nicotinic receptors, pharmacological chaperoning increases ER exit sites (ERES), which is a key mechanism used by the cell to transport proteins from the ER to the Golgi [34, 35]. Sec24 is a key component of the ERES and has been used to mark ERES in cellular studies. Additionally, these prior studies have shown that an increase in ERES leads to an increase in the surface expression of the plasma membrane protein. Here, we establish that Sec24D co‐localizes in the ER with MORs, suggesting that Sec24D shuttles MORs. We used Sec24D‐eGFP to visualize ERES and observe changes in the fraction of the cytoplasm that ERES occupy in response to drug treatment. Heinzer et al. reasoned that increasing the size of ERES is a more advantageous strategy for increasing secretory flux than increasing the number of ERES, thus measuring the fraction of the cytoplasm occupied by ERES is more appropriate than measuring the total number of ERES [36]. The possibility that antagonists induce supersensitivity by pharmacologically chaperoning opioid receptors could suggest innovative approaches for opioid abuse disorder.

Additionally, we tested several other MOR ligands. Interestingly, we found that morphine and fentanyl, but not buprenorphine, methadone, or two allosteric modulators, also increase ERES levels. Finally, we tested the MOR[N190K][S375A] mutant since the S375 is an important phosphorylation site following MOR activation [37, 38, 39, 40]. Interestingly, this mutant was pharmacologically chaperoned by Ntx, but not by morphine or fentanyl, suggesting a broader role for the S375 residue. Similar to other pharmacological chaperoning events, we found that inhibiting retrotransport from the Golgi prevented Ntx or naloxone from acting as pharmacological chaperones. Finally, we found no evidence that [cAMP] increases with Ntx treatment, suggesting that Ntx does not increase global protein trafficking. Altogether, these results yield new insights into how opioid ligands act on opioid receptors.

## 2 Materials and Methods

### 2.1 Reagents

All reagents were obtained from Sigma‐Aldrich (St. Louis, MO). The MOR plasmid was a gift from Dr. Brigitte Kieffer. The MOR‐mCherry plasmid was purchased from VectorBuilder (The vector ID is VB181109‐1086uuu, which can be used to retrieve detailed information about the vector on vectorbuilder.com). The eGFP‐Sec24D plasmid was used as previously described. All other mutants were generated using site‐directed mutagenesis following standard protocols.

### 2.2 SH‐SY5Y Cell Culture and Transfection

SH‐SY5Y cells were purchased from the ATCC (CRL‐2266) (RRID:CVCL_0019). Cells were cultured according to the protocols specified by the ATCC, except Opti‐Mem was used instead of EMEM/F12. Mycoplasma contamination was assayed at 6‐month intervals and remained negative throughout these experiments. Cells were cultured in 35 mm dishes with a glass coverslip on the bottom and allowed to reach 90% confluency before transfection. Transfection was carried out using Lipofectamine 3000 (Thermo Fisher) using the standard protocols. Typical DNA loads were between 0.5 and 1.0 μg per dish. Transfection media was replaced 24 h post‐transfection with growth media. Drugs were added to the cell media to give a total drug concentration of 10 μM, 12 h before imaging. Before imaging, the cells were washed once with phosphate‐buffered saline (PBS) and then remained in fresh PBS for imaging.

### 2.3 Z‐Stack Confocal Microscopy

The Zeiss LSM 880 with “Fast Airyscan” was used. The pixel dwell time was ~0.2 μs. Each slice was taken 0.1 μm from the previous one. All images and z‐stacks were processed using Airyscan processing in the Zen Blue software package (Zeiss).

### 2.4 Sensitized Emission Förster Resonance Energy Transfer (FRET)

FRET experiments were performed following procedures described by Elder and co‐workers [41] and previously used in this lab [42, 43] to calculate sensitized emission FRET with corrections for donor bleed‐through into the acceptor channel and vice versa. SH‐SY5Y cells were transfected with either Sec24D‐eGFP, Rab5‐eGFP, or MOR[N190K]‐mCherry to establish the acceptor emission ratio (AER) or donor emission ratio (DER). These ratios were calculated using the equations:

(1) $$\mathrm{AER}=\frac{I^{\mathrm{AD}}}{I^{\mathrm{AA}}}$$

(2) $$\mathrm{DER}=\frac{I^{\mathrm{DA}}}{I^{\mathrm{DD}}}$$

where *I* ^*AD*^ is mCherry fluorescence intensity in the mCherry channel with 488 nm excitation, *I* ^*AA*^ is mCherry fluorescence intensity in the mCherry channel with 561 nm excitation, *I* ^*DA*^ is eGFP fluorescence intensity in the mCherry channel with 488 nm excitation, and *I* ^*DD*^ is eGFP fluorescence intensity in the eGFP channel using 488 nm excitation. The FRET samples were transfected with MOR[N190K]‐mCherry and Sec24D‐eGFP or Rab5‐eGFP. Using the AER and DER values calculated in the samples expressing one fluorescent protein, the following equation was used to calculate the corrected FRET intensity, cFRET.

(3) $$\text{cFRET}=I^{\mathrm{DA}}-\left(\mathrm{DER}\right)\left(I^{\mathrm{DD}}\right)-\left(\mathrm{AER}\right)\left(I^{\mathrm{AA}}\right)$$

where *I* ^*DA*^, *I* ^*DD*^, and *I* ^*AA*^ are measured in the FRET sample using the same parameters as those used in the samples to obtain the AER and DER. Using the cFRET value, we calculated FRET efficiency as follows:

(4) $$\text{FRET Efficiency}=\frac{\text{cFRET}}{I^{\mathrm{AA}}}$$

All five samples were imaged on the same day on the same microscope.

### 2.5 Image Analysis

All 2D image analysis was performed using FIJI. To determine the threshold to designate ERES, the 2D image was smoothed three times in FIJI and an ROI was drawn around the entire cell. The top 5% of the most intense pixels that constitute a puncta larger than 0.1 μm^2^ in the ROI were marked as ERES. This same intensity threshold was used to designate ERES in the 3D images which were analyzed in Imaris (Bitplane). Among the various metrics presented by Imaris, we emphasize the fraction of cytoplasmic volume occupied by ERES, because this volume‐integrated metric is relatively insensitive to assumptions about size, shape, and number of individual particles.

### 2.6 Competitive ELISA for [cAMP] Measurement

SH‐SY5Y cells were grown to 90% confluency and transfected with MOR[N190K]. The cells were lysed with 0.1 M HCl 48 h post‐transfection and after the appropriate drug treatment (12 h with 10 μM Ntx, 15 min with 10 μM forskolin (positive control for [cAMP] upregulation), or vehicle [water]). Lysates were centrifuged at 15000 rpm for 10 min at 4°C on a standard table‐top centrifuge to remove insoluble debris. The supernatant was used for the remainder of the procedure as described by the protocol provided with the cAMP Assay Kit (Abcam, ab65355).

### 2.7 Statistical Analysis

All results are presented as mean ± S.E.M. and all statistical analyses were performed using GraphPad Prism. Statistical significance was determined by an unpaired Student's *t*‐test (in comparisons between two samples) or an ANOVA with a post hoc Tukey test (in comparison's with more than two samples). In comparisons where the *p*‐value < 0.05, these differences were considered significant.

## 3 Results

### 3.1 MOR[N190K] Reaches Wild‐Type Plasma Membrane Densities After Ntx or Naloxone Treatment

**FIGURE 1:**
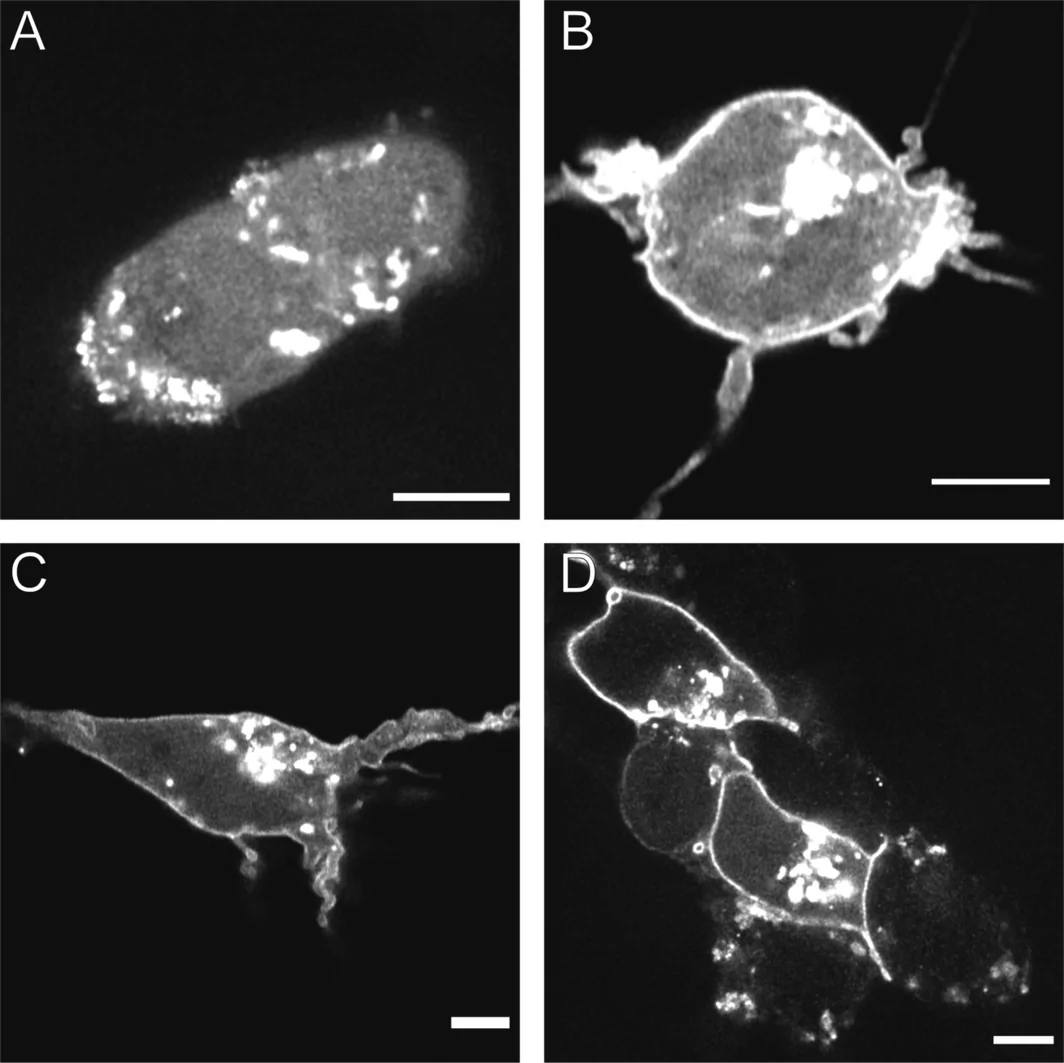
MOR[N190K] appears on the plasma membrane after 24 h incubation in 10 μM Ntx or naloxone. Representative images of SH‐SY5Y cells transfected with MOR[N190K]‐mCherry and treated with (A) vehicle, (B) 10 μM Ntx, or (C) 10 μM naloxone for 12 h. The vehicle‐treated samples show no plasma membrane localization of MOR[N190K]‐mCherry while both the Ntx and naloxone treated cells are outlined with fluorescent signal, suggesting an increase in plasma membrane localization. (D) SH‐SY5Y cell overexpressing wild‐type MOR‐mCherry. Scale bars are 5 μm.

In contrast to the other opioid receptor subtypes, MORs are primarily localized on the plasma membrane [4]. This lack of ER‐localized MORs vitiates systematic studies of chaperoning. To increase the pool of MORs in the ER, we generated the MOR[N190K] mutant [33]. This N190K mutation impairs trafficking: most MOR[N190K] is retained in the cytoplasm. Fortin and co‐workers state that wild‐type plasma membrane expression is rescued by treating the MOR[N190K] expressing cells with Ntx or naloxone [33]. Here, we generated the MOR[N190K] mutant with mCherry on the C‐terminus to visualize the receptor. After antagonist treatment, we did observe fluorescent intensity outlining the cell, resembling the localization pattern of the wild‐type MOR, suggesting an increase in plasma membrane localization compared to vehicle‐treated cells (Figure 1). These results are consistent with Fortin and co‐worker's conclusions.

### 3.2 MOR Interacts With Sec24D

**FIGURE 2:**
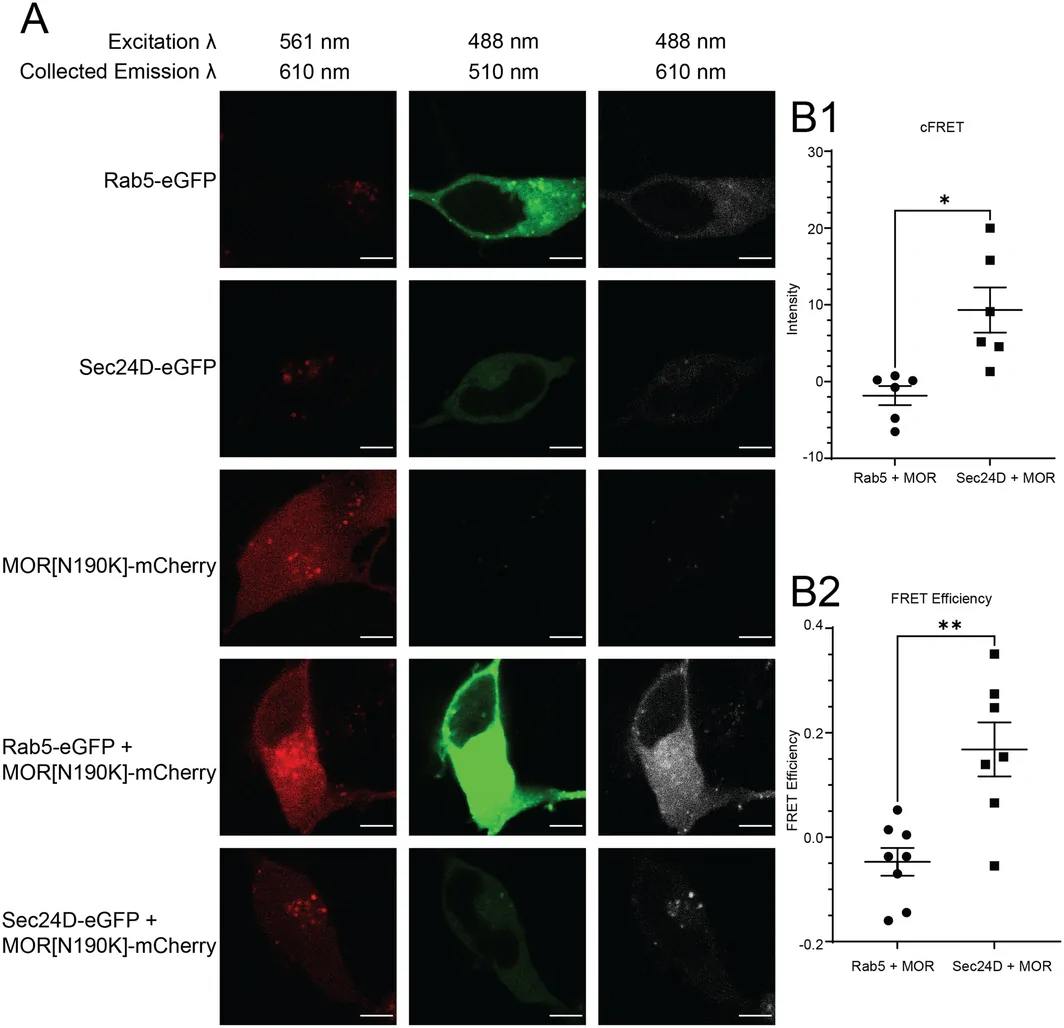
FRET analysis shows that in living cells, MOR is close to Sec24D. (A) Representative images of SH‐SY5Y cells expressing Rab5‐eGFP, Sec24D‐eGFP, MOR[N190K]‐mCherry, Rab5‐eGFP and MOR[N190K]‐mCherry, or Sec24D‐eGFP and MOR[N190K]‐mCherry. Left column, samples are excited at 561 nm excitation filter and imaged at 610 nm to reveal mCherry. Center column, samples are excited with at 488 nm excitation filter and imaged at 510 nm to reveal eGFP. Right column, samples are excited at 488 nm excitation filter and imaged at 610 nm to reveal sensitized emission. (B1) The cFRET intensities and (B2) FRET efficiency from the two FRET samples (Rab5‐eGFP and MOR[N190K]‐mCherry or Sec24D‐eGFP and MOR[N190K]‐mCherry). *p*‐values are calculated from a student's *t*‐test. *, *p* < 0.05; ***, *p* < 0.0005. Scale bar is 5 μm.

There are four Sec24 isoforms, so it is imperative to validate that a specific isoform interacts with the MOR. We hypothesize that Sec24D is the most important isoform as this isoform has been used in previous pharmacological chaperoning studies [34, 44]. In order to verify that the MOR and Sec24D interact with each other, we performed sensitized emission FRET experiments [41]. The donor was eGFP attached to Sec24D and the acceptor was mCherry attached to MOR[N190K]. The MOR[N190K] mutant was used to ensure that a large pool of receptors would be in the ER and available to interact with Sec24D. As a negative control, we used Rab5‐eGFP as a donor. Rab5 localizes to early endosomes, thus we would not expect Rab5 to interact extensively with the MOR[N190K] [45]. This control will also ensure that we are not observing receptors in the early endosome, which would instead test the recycling hypothesis. The MOR[N190K]‐mCherry + Rab5‐eGFP samples serve as an additional negative control to rule out spectral bleed‐through from the donor excitation wavelength to the acceptor excitation spectrum. We calculated cFRET, a sensitized emission FRET method that corrects for spectral bleed‐through from the donor excitation to acceptor emission in the absence of FRET (see Methods). We found that the Sec24D‐MOR[N190K] FRET pair results in significantly greater cFRET intensity and FRET efficiency than the Rab5‐MOR[N190K] FRET pair (Figure 2). These results suggest that the MOR interacts with Sec24D.

### 3.3 Ntx, Naloxone, Morphine, and Fentanyl Induce Increases in ERES in SH‐SY5Y Cells Overexpressing MOR[N190K]

**FIGURE 3:**
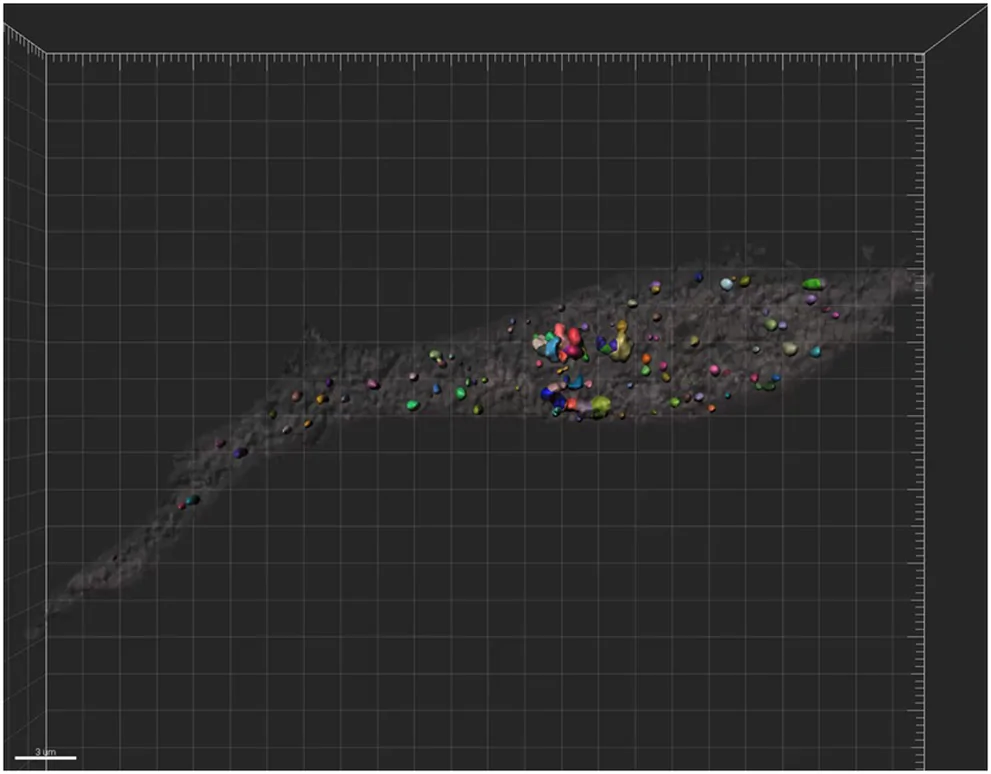
Representative image of a 3D rendering of the z‐stacks obtained for ERES analysis. The image is a screenshot of Movie 1. Each slice of the z‐stack is 0.1 μm apart. The transparent gray region represents the cytoplasm. The opaque structures represent ERES. Each color represents a separate ERES. The analysis distinguishes individual ERES within a cluster, based on the assumption measurements that the average ERES is 500 nm in diameter (Heinzer et al.). However, this distinction is not used to calculate the fraction of cytoplasm occupied by ERES. Scale bar is 3 μm.

**FIGURE 4:**
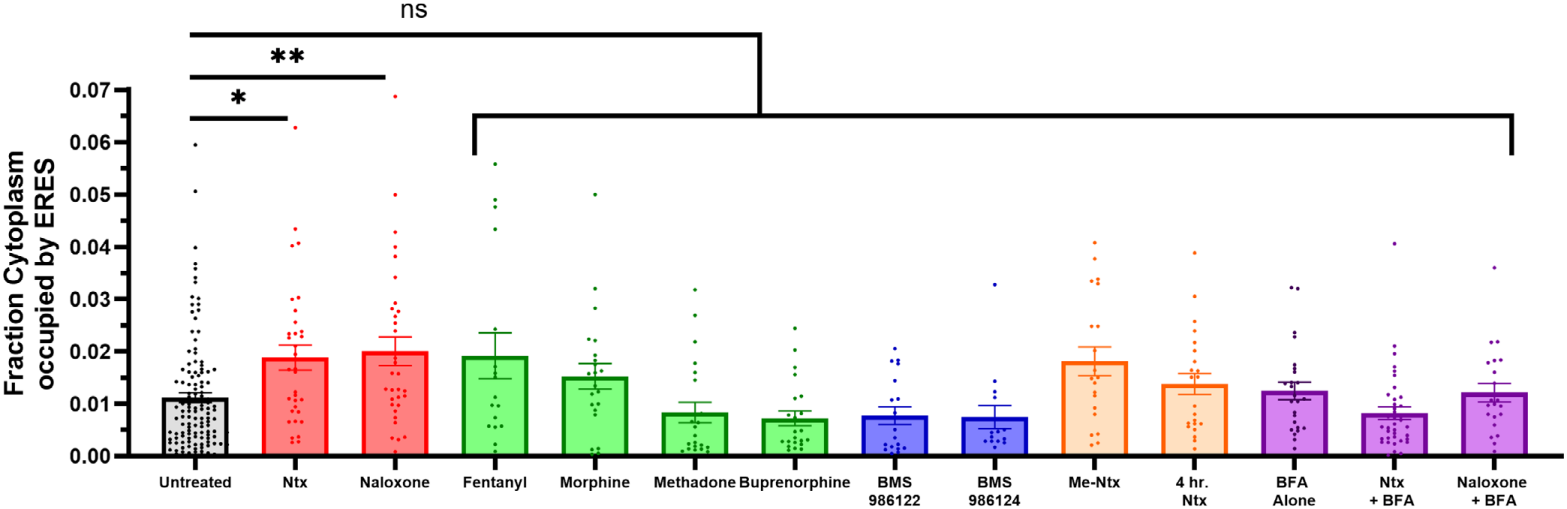
ERES levels are higher after Ntx, Me‐Ntx, naloxone, morphine, or fentanyl exposure. All incubation times are 12 h at 10 μM concentration in SH‐SY5Y cells transfected with MOR[N190K] and Sec24D‐eGFP. The black data are untreated samples (*n* = 127). The red bars are cells treated with an antagonist (Ntx [*n* = 33] or naloxone [*n* = 32]). The green bars represent cells treated with an agonist (fentanyl [*n* = 17], morphine [*n* = 22], methadone [*n* = 22], or buprenorphine, [*n* = 22]). The blue bars represent cells treated with an allosteric modulator (BMS 986122 [*n* = 18] or BMS 986124 [*n* = 14]). The orange bars represent cells treated with either a modified Ntx (Me‐Ntx) (*n* = 20) or Ntx with a briefer incubation (4 h, *n* = 23). The purple bars represent cells treated with a COPI inhibitor, brefeldin A (BFA) (BFA Alone, *n* = 25) (Ntx + BFA, *n* = 38) (Naloxone + BFA, *n* = 21). *p*‐values are calculated from an ANOVA and post hoc Tukey test comparing to the untreated cells. *, *p* < 0.05; **, *p* < 0.01; ns, *p* > 0.05.

To observe ERES specifically upregulated due to MOR chaperoning, we overexpress the ER‐retained MOR mutant, MOR[N190K], in SH‐SY5Y cells [33]. We used SH‐SY5Y cells as they have been used extensively to understand the cell biology of mental disorders and we are most interested in MOR trafficking in the context of opioid use disorder [46, 47, 48, 49, 50, 51, 52]. The percentage of the cytoplasm occupied by ERES was measured in SH‐SY5Y cells transfected with MOR[N190K] and Sec24D‐eGFP after 12 h incubation in 10 μM of an opioid ligand. We chose to analyze the z‐stacks to investigate ERES volume in the entire cell (Figure 3 and Movie 1). Our analysis suggests that Ntx, naloxone, morphine, and fentanyl increase ERES levels. In contrast, buprenorphine, methadone, and the allosteric modulators did not increase ERES levels (Figure 4).

### 3.4 N‐Methyl‐Naltrexone Causes a Modest Shift in ERES Levels

To test the dependence of antagonist entry into the cytoplasm for ERES level increases, we used N‐methyl‐naltrexone (Me‐Ntx), a permanently charged derivative of Ntx [53]. Me‐Ntx should cross the plasma membrane at a substantially lower rate than Ntx. Since Me‐Ntx should not reach the ER, the pharmacological chaperoning hypothesis predicts that Me‐Ntx would not induce as strong a rise in ERES levels. Indeed, we observe a less substantial shift in ERES levels following Me‐Ntx treatment in SH‐SY5Y cells overexpressing MOR[N190K] (Figure 4). These results suggest that the general hydrophobic nature of Ntx is sufficient to allow it to cross the plasma membrane and induce increases in ERES levels.

### 3.5 Temporal Dependence of ERES Level Increases Following Ntx Treatment

Next, we tested the temporal dependence of the ERES upregulation event. Observing the increase in MORs on the surface typically requires ~24 h incubation with the antagonist to reach peak MOR surface densities [54]. We also tested 4 h incubations with Ntx and only observed a partial increase in ERES levels, suggesting that some pharmacological chaperoning may be occurring at this time, but not as much after 12 h treatment (Figure 4).

### 3.6 Mutating S375 Prevents Morphine and Fentanyl Induced Pharmacological Chaperoning

**FIGURE 5:**
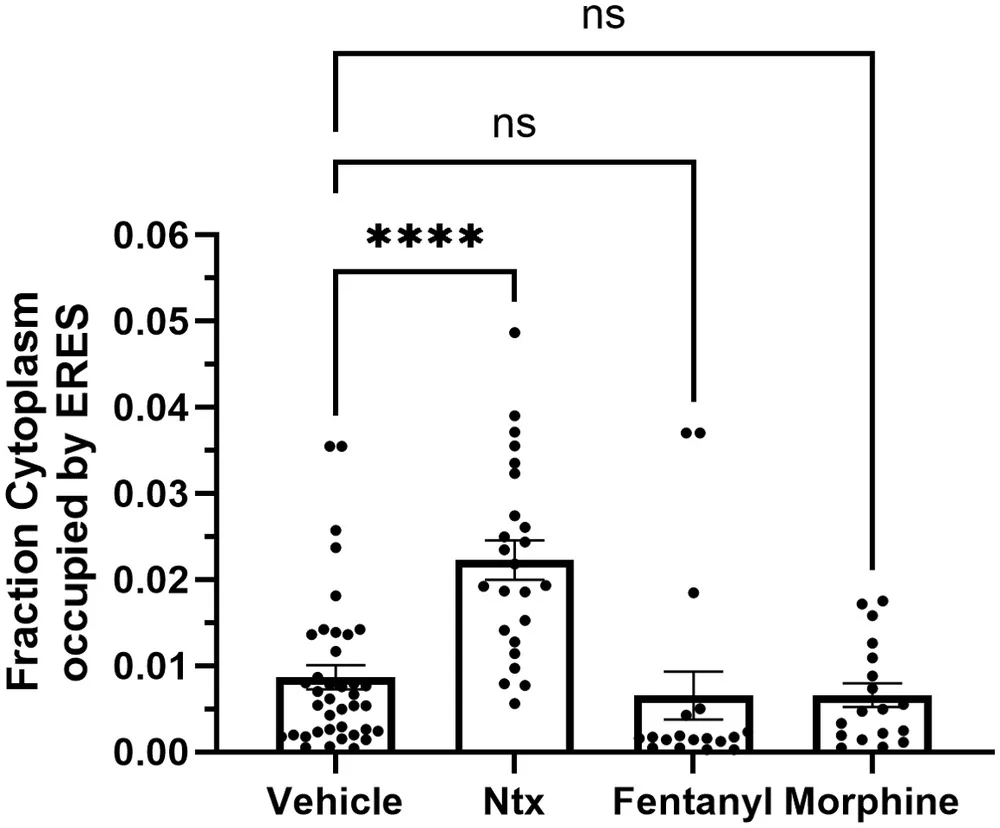
Ntx but not agonists increase ERES levels in SH‐SY5Y cells in SH‐SY5Y cells transfected with MOR[N190K][S375A] and Sec24D‐eGFP. *p*‐values are calculated from an ANOVA and post hoc Tukey test. ****, *p* < 0.0001; ns, *p* > 0.05.

When MORs become activated, they undergo a series of phosphorylation events before arrestin recruitment and endocytosis [55]. One of the key residues for phosphorylation is S375: point mutations at this site are most effective for reducing receptor internalization [37, 38, 39]. We hypothesized that the S375 residue might also influence MOR's trafficking from the ER as it is possible that agonists are able to induce S375 phosphorylation away from the plasma membrane. Ntx continues to induce a rise in ERES levels in SH‐SY5Y cells overexpressing MOR[N190K][S375A], which is consistent with the inability of Ntx to induce S375 phosphorylation (Figure 5). However, morphine and fentanyl do not increase ERES levels in SH‐SY5Y cells overexpressing MOR[N190K][S375A](Figure 5). These results suggest that S375 may be an important residue for morphine and fentanyl to pharmacologically chaperone the MORs. While it is not clear whether S375 is becoming phosphorylated by agonists while the MOR matures in the ER, mutating this residue to alanine appears to abrogate the chaperoning effects of agonists.

### 3.7 Ntx Does Not Induce a Rise in [cAMP]

**FIGURE 6:**
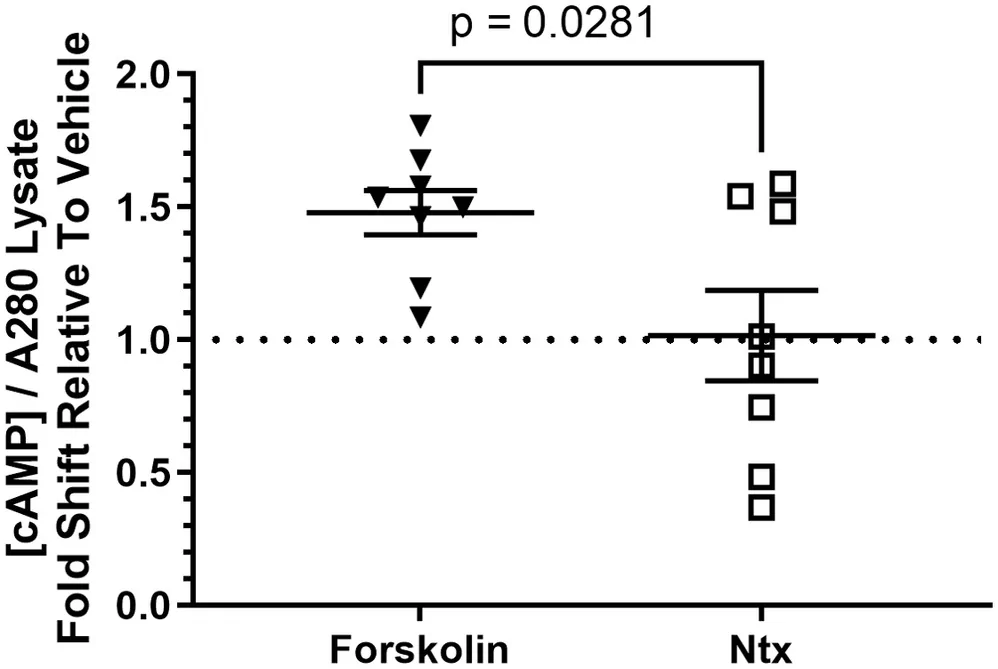
[cAMP] levels do not change in cells treated with Ntx. SH‐SY5Y cells were transfected with MOR[N190K] as in ERES imaging experiments. These cells were then treated with either 10 μM Ntx for 12 h, vehicle for 12 h followed by 10 μM forskolin for 15 min, or vehicle for 12 h. Forskolin‐treated cells serve as a positive control for [cAMP] increases. Measurements for [cAMP] were normalized to vehicle‐treated samples (represented by the dotted line). These results show that [cAMP] increases in forskolin‐treated but not in Ntx‐treated cells, relative to vehicle‐treated samples. *p*‐values are calculated from an unpaired Student's *t*‐test where *p* < 0.05 is considered significant for the ELISA assays.

An increase in intracellular [cAMP] occurs when cargo export increases in fibroblasts or HeLa cells [22]. It is not known whether there is an increase in [cAMP] with Ntx treatment without prior MOR activation. Here we tested whether Ntx increases [cAMP] in SH‐SY5Y cells overexpressing MOR[N190K]. An ELISA competition assay was used to measure relative concentrations of cAMP in SH‐SY5Y cells transfected with MOR[N190K] after 12 h incubation with 10 μM Ntx, 15 min incubation with 10 μM forskolin (positive control for increases in [cAMP]), or vehicle. Our results suggest that Ntx does not increase [cAMP] (Figure 6). This is not surprising because the most likely way for Ntx to increase [cAMP] is by further inhibiting the MOR below its basal activity. Observing MOR activity typically requires activation with agonists; thus, observing a significant increase in [cAMP] by further inhibiting MORs is unlikely [56, 57]. Since cAMP concentration changes have been ruled out as the cause of MOR upregulation, the chaperoning hypothesis remains the leading explanation for how Ntx and naloxone increase ERES levels.

### 3.8 Ntx Pharmacological Chaperoning Depends on COPI

Coat protein I (COPI) vesicles [58, 59, 60] retrotransport cargo from the Golgi to the ER to aid in sorting and protein quality control [61]. COPI vesicles are composed of seven core subunits, α‐COP, β’‐COP, ε‐COP, βCOP, δ‐COP, γ‐COP, and ζ‐COP and are thus distinct from ERES. Prior work in our lab and others has demonstrated that COPI vesicles can play a role in pharmacological chaperoning [24, 62]. Here, we find a similar dependence as Ntx and naloxone no longer increase ERES levels if the cells are co‐treated with brefeldin A (BFA), a COPI inhibitor (figure 4, purple) [24, 54, 63]. These results suggest that some of the ERES observed in the non‐BFA treated cells have already traveled to the Golgi. This suggests that MORs must undergo multiple rounds of ER‐Golgi trafficking before final maturation and presentation on the plasma membrane. This retrograde trafficking dependence is not uncommon for some cargos, and it appears that the MORs also depend on some ER‐Golgi cycling before they are ready to go to the plasma membrane.

## 4 Discussion

Although there are numerous efforts to induce analgesia by pharmacologically targeting voltage‐gated sodium channels and some nicotinic receptors, at present, the MOR continues to be the primary target for analgesic drugs [64, 65]. As a plasma membrane protein, MOR trafficking is important to its function and thus plays a prominent role in opioid use disorder. If we can better understand MOR trafficking and manipulate MOR surface densities in predictable ways, we may better treat opioid use disorder. Until now, there have been no studies done on ERES and MORs.

Here we show that for a mutant MOR that builds to observable levels in the ER, Ntx, naloxone, morphine, and fentanyl increase the fraction of the cytoplasm occupied by ERES (Figure 4). This is likely an early event in the pathway that results in increased opioid receptor surface densities and supersensitivity following antagonist exposure. However, increased opioid receptor surface densities do not follow morphine or fentanyl treatment, likely because the competing internalization pathways prevail and result in a net decrease in opioid receptor surface densities. By demonstrating that this adverse effect of antagonist treatment is manifested through ERES and that morphine and fentanyl also increase MOR trafficking, we show that ERES is a critical component in the function of opioid receptors.

S375 phosphorylation is a key event during receptor activation [37, 39]. We decided to see if this residue is important to the chaperoning capabilities of Ntx, morphine, or fentanyl. To test this, we made MOR[N190K][S375A], a MOR mutant that is highly‐ER retained and cannot be phosphorylated at S375. Interestingly, while Ntx remained a strong MOR pharmacological chaperone, morphine and fentanyl did not induce any increases in ERES levels (Figure 5). The results from tests with MOR[N190K] and MOR[N190K][S375A] suggest that S375 is important for the pharmacological chaperoning done by morphine and fentanyl. Furthermore, these results suggest an internal role for S375. While most studies have emphasized the importance of S375 phosphorylation in receptor internalization, we find that its influence may also be observed at the ER.

We ruled out that the increases in ERES arise from increases in [cAMP] by measuring [cAMP] in MOR[N190K]‐overexpressing SH‐SY5Y cell lysates after incubation in Ntx, vehicle, or forskolin using a competitive ELISA assay. Here, we overexpressed MOR[N190K] to most closely replicate cells used in the imaging experiments. SH‐SY5Y cells endogenously express the MOR, thus there will be MORs exhibiting basal activity at the plasma membrane that could be reduced by antagonist exposure [46]. Despite decreasing the basal activity of the MORs, we did not observe a significant change in [cAMP] in cells treated with Ntx (Figure 6). Since [cAMP] does not increase during Ntx treatment, the cAMP hypothesis is not supported and thus provides further support for the chaperoning hypothesis.

Our work does not challenge the prevailing view that, for the wild type MOR, agonists affect MOR via endocytosis at the plasma membrane—a much later stage than studied here [66]. Interestingly, buprenorphine does not increase ERES levels, although Fortin and co‐workers found that they could rescue MOR[N190K] plasma membrane expression with buprenorphine [33]. These two observations suggest that buprenorphine increases MOR surface expression in an ERES‐independent fashion. Indeed, there are several trafficking steps that can be modulated by small molecules [67, 68, 69]. Pathways to the plasma membrane vary among MORs, and one may target one route over the other [70, 71, 72]. Our results, along with Fortin and co‐worker's data, suggest that buprenorphine affects MOR trafficking using a different mechanism than Ntx or naloxone.

These results also give insight into how the mutant MOR matures in the ER and Golgi. The lack of ERES increases with COPI inhibition suggests some recycling between the ER and the Golgi. This observation is consistent with the incubation times with naltrexone required before observing increased MOR plasma membrane levels [54]. We also observe some time dependence with our ERES experiments (Figure 4). We hypothesize that after 4 h, the mutant MOR has not had an opportunity to enter the Golgi‐ER pathway fully, so we see further increases in ERES levels following 12 h treatment. Furthermore, these COPI experiments are consistent with prior reports demonstrating the importance of retrograde transport for pharmacological chaperoning [24, 62].

Our results provide key information regarding MOR trafficking. Our work also continues to make a case for pharmacological chaperoning as an important part of a drug's efficacy profile [28]. This principle of “inside‐out pharmacology” involves appreciating the fact that ligands can increase the trafficking of receptors, thus changing how a cell or animal may respond to ligand exposure [73]. Furthermore, we find an “inside‐out” role for the S375 residue, a site mainly studied for its effects on plasma membrane MORs.

Our study applies only to the rare MOR[N190K] mutant; in pilot experiments, we found neither upregulation nor effects on ERES levels with wild‐type MOR. This limits our ability to explain the mechanism of upregulation/supersensitivity of MOR found in studies on intact animals with WT MORs treated with antagonists [14, 74, 75]. The microscopy experiments like those described here cannot yet be applied to native neurons of intact neural pathways.

One would need to invoke additional, unknown aspects of chaperoning to generalize its usefulness as a mechanism for upregulation/supersensitivity. This point resembles the uncertainties about mechanisms that allow nicotine to upregulate nicotinic receptors of only some cell types and only at some subcellular regions [73].

Ultimately, our work here shows that several opioid ligands, both agonists and antagonists, affect the internal processing of MORs. Although most of the functions of MORs occur at the plasma membrane, our results suggest that ligands impact the trafficking of these receptors in ways that ultimately impact their function on the plasma membrane. As we have observed with nicotinic receptors, the “inside‐out pharmacology” appears to be a phenomenon across several drugs of abuse and warrants further exploration. We hope that our work inspires further experiments that investigate the impact of opioid ligands on opioid receptors away from the plasma membrane.

## Author Contributions

**Matthew J. Mulcahy:** investigation, writing – original draft, writing – review and editing, methodology. **Henry A. Lester:** resources, supervision, formal analysis, project administration, writing – review and editing, visualization, methodology, conceptualization, investigation, funding acquisition, writing – original draft. **Stephen N. Grant:** conceptualization, investigation, funding acquisition, writing – original draft, writing – review and editing, methodology, formal analysis, validation.

## Funding

Funding was provided by the National Institutes of Health (NIH) DA036061, DA037161, and DA037743 (HAL); NIH DA046122 (SNG). The content is solely the responsibility of the authors and does not necessarily represent the official views of the National Institutes of Health.

## Ethics Statement

The authors have nothing to report.

## Conflicts of Interest

The authors declare no conflicts of interest.

## Supporting information


**Movie 1:** Representative 3D rendering of the combined z‐stacks from a cell used to perform ERES analysis. The transparent region represents the cytoplasm, and the opaque structures are ERES (ERES determination is described in the methods). Each color represents a different ERES, based on the assumption that the ERES is a maximum of 500 nm in diameter. However, the differentiation of ERES is not used in our analysis. Scale bar: 3 μm.

## Data Availability

The data that support the findings of this study are available from the corresponding author upon reasonable request.
